# From the gene to the clinic: Key milestones in the PI3Kδ journey

**DOI:** 10.1093/jimmun/vkag075

**Published:** 2026-05-19

**Authors:** Bart Vanhaesebroeck

**Affiliations:** UCL Cancer Institute, University College London, London, United Kingdom

**Keywords:** PI3K, PI 3-kinase, signaling, cancer, leukemia, lymphoma, drug, medicine

## Abstract

Among the PI3K isoforms, PI3Kδ has attracted particular attention from immunologists and hematologists. Despite earlier clinical setbacks, the PI3Kδ field has recently re-emerged with renewed promise. Here, I highlight key milestones in the evolving understanding of PI3Kδ signaling in immunity and cancer, and pivotal studies that have shaped this field.

In this mini-review, I chart the key milestones that have shaped our understanding of PI3Kδ, presented as a timeline in Figure  1.

**Figure 1:**
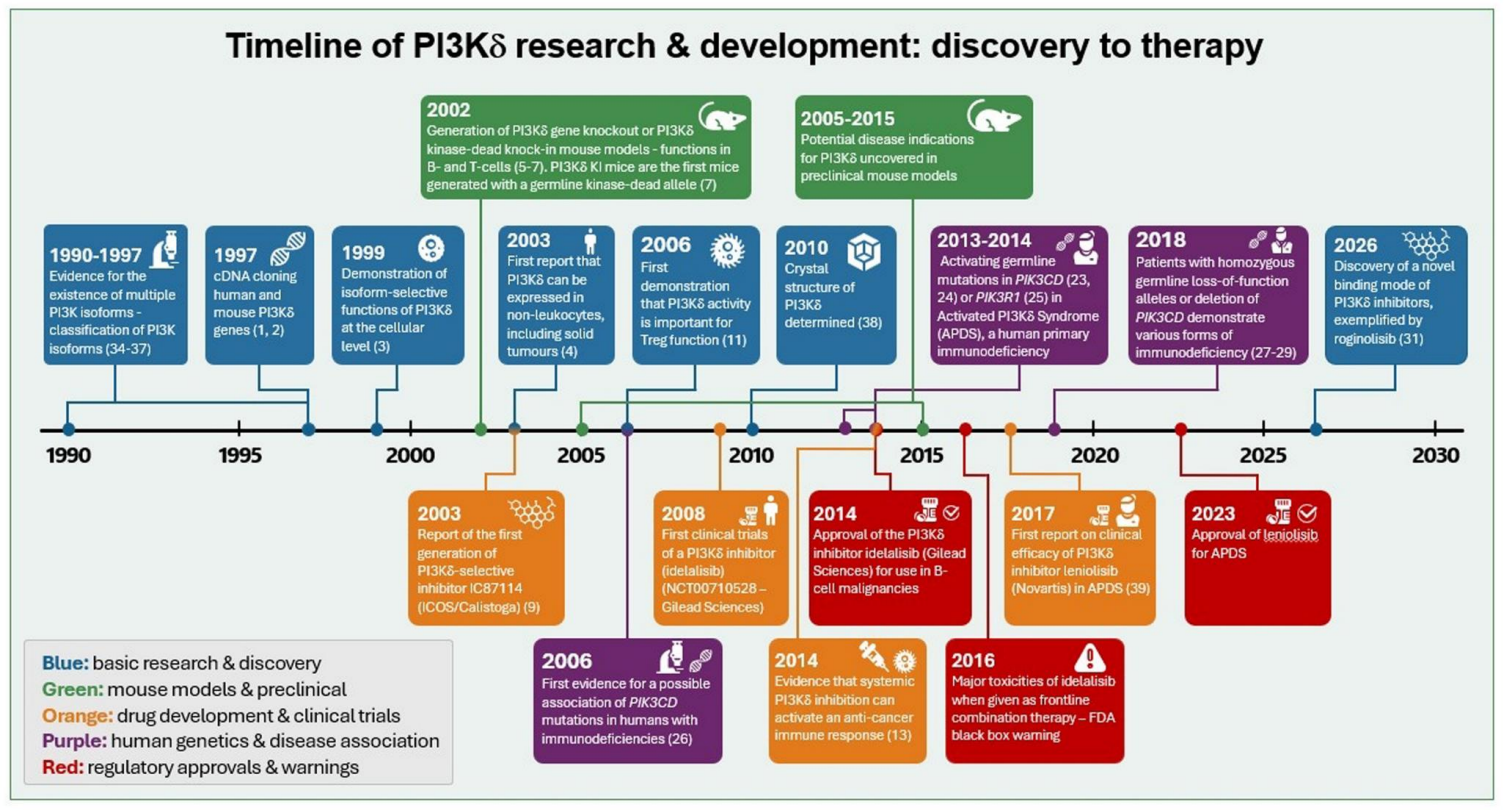
Timeline of key discoveries concerning PI3Kδ. (1) 1990–1997: Evidence for the existence of multiple PI3K isoforms—classification of PI3K isoforms.^34–37^ (2) 1997: Complementary DNA (cDNA) cloning human and mouse PI3Kδ genes.^1^^,^^2^ (3) 1999: Demonstration of isoform-selective functions of PI3Kδ at the cellular level.^3^ (4) 2002: Generation of PI3Kδ gene knockout or PI3Kδ kinase-dead knock-in mouse models–functions in B and T cells.^5–7^ PI3Kδ KI mice are the first mice generated with a germline kinase-dead allele.^7^ (5) 2003: First report that PI3Kδ can be expressed in nonleukocytes, including solid tumors.^4^ (6) 2003: Report of the first generation of PI3Kδ-selective inhibitor IC87114 (ICOS/Calistoga).^9^ (7) 2005 to 2015: Potential disease indications for PI3Kδ uncovered in preclinical mouse models. (8) 2006: First evidence for a possible association of *PIK3CD* mutations in humans with immunodeficiencies.^26^ (9) 2006: First demonstration that PI3Kδ activity is important for Treg function.^11^ (10) 2008: First clinical trials of a PI3Kδ inhibitor (idelalisib) (NCT00710528—Gilead Sciences). (11) 2010: Crystal structure of PI3Kδ determined.^38^ (12) 2013 to 2014: Activating germline mutations in *PIK3CD*^23^^,^^24^ or *PIK3R1*^25^ in APDS, a human primary immunodeficiency. (13) 2014: Approval of the PI3Kδ inhibitor idelalisib (Gilead Sciences) for use in B cell malignancies. (14) 2014: Evidence that systemic PI3Kδ inhibition can activate an anticancer immune response.^13^ (15) 2018: Patients with homozygous germline loss-of-function alleles or deletion of *PIK3CD* demonstrate various forms of immunodeficiency.^27–29^ (16) 2016: major toxicities of idelalisib when given as frontline combination therapy—FDA black box warning for the clinical use of idelalisib. (17) 2017: First report on clinical efficacy of PI3Kδ inhibitor leniolisib (Novartis) in APDS.^39^ (18) 2023: Approval of leniolisib for APDS. (19) 2026: Discovery of a novel binding mode of PI3Kδ inhibitors, exemplified by roginolisib.^30^

In 1997, we and others reported the isolation of the complementary DNA for the human and mouse p110δ catalytic subunit of PI3Kδ, encoded by the *PIK3CD* gene.^1^^,^^2^ Compared with the ubiquitously expressed PI3Kα and PI3Kβ, an early distinguishing feature of PI3Kδ was its highly enriched expression in leukocytes,^1^^,^^2^ suggestive of a possible role in immune signaling. Microinjection studies using neutralizing PI3K antibodies in cell-based models provided some of the earliest evidence for isoform-selective, nonredundant roles of PI3Kα, PI3Kβ, and PI3Kδ in leukocytes.^3^ Beyond white blood cells, we showed that PI3Kδ can also become highly expressed in certain solid tumors, such as melanoma^4^ in which its functions remain unknown.

An immunological role for PI3Kδ was formally revealed in 2002 through the characterization of PI3Kδ gene–knockout and PI3Kδ kinase–dead mouse models.^5–7^ While all 3 studies investigated B cells, our group also characterized T cell phenotypes.^7^ In both B and T lymphocytes, PI3Kδ was found to be particularly important in signaling downstream of the antigen receptors.^5–7^ We subsequently also demonstrated a role for PI3Kδ for the FcεRI antigen receptor in mast cells,^8^ a finding exploited in the in vitro diagnostic BASOTEST readout for PI3Kδ, which measures FcεRI-induced basophil degranulation in whole blood by flow cytometry through the detection of surface activation markers such as CD63.

PI3Kδ gene–targeted mice show largely intact early B cell development but display a reduction in marginal zone B cells and B1 cell subsets, with impaired T cell–dependent and T cell–independent humoral immune responses.^5–7^ T cell development is largely intact, but antigen-specific T cell responses are reduced.^7^ These mouse gene–targeting studies raised considerable interest in PI3Kδ as a target for autoimmune and inflammatory indications, as well as in B cell malignancies, prompting several companies to pursue PI3Kδ inhibitor development. An early adopter of this effort was ICOS Corporation which in 2003 reported IC87114, a first-generation PI3Kδ-selective inhibitor.^9^ Together with PI3Kδ gene–targeted mice, this compound greatly facilitated preclinical studies on PI3Kδ biology.

Such studies allowed a detailed characterization of the biological functions of PI3Kδ, which was found to be the functionally dominant PI3K isoform in lymphocytes. This contrasts with PI3Kγ, the other leukocyte-enriched PI3K isoform, which plays a more important role in myeloid cells, although this distinction is not absolute. For example, PI3Kδ can also play a role in dendritic cells where it is a negative regulator of Toll-like receptor signaling.^10^

PI3Kδ kinase–dead mice also develop gut inflammation,^7^ an observation later shown to be due to a defect in regulatory T cells (Tregs)^11^ and mirrored by colitis in humans treated with PI3Kδ inhibitors.^12^ Our study in 2014 further showed that Treg inhibition by systemic PI3Kδ inhibition can activate a host anticancer adaptive immune response,^13^ which is the basis for the ongoing cancer immunotherapy trials described subsequently.

In 2006, Calistoga Pharmaceuticals was founded as a spin-off of the PI3Kδ inhibitor drug development program from ICOS, shortly before ICOS was acquired by Eli Lilly. Calistoga’s lead asset was CAL-101 (idelalisib), which in phase 2 trials demonstrated significant clinical activity in patients with relapsed chronic lymphocytic leukemia (CLL)^14^ and relapsed indolent non-Hodgkin’s lymphoma.^15^ In fact, following early termination owing to strong efficacy, patients in the placebo arm were permitted to cross over to idelalisib. In 2011, Calistoga was acquired by Gilead Sciences, who in 2014 gained Food and Drug Administration (FDA) approval for idelalisib (trade name Zydelig) for CLL, follicular lymphoma, and small lymphocytic lymphoma.

Given that PI3Kδ inhibitors do not have clear cytotoxic activity for leukemic cells in vitro, the robust therapeutic benefit in phase 2 trials was somewhat surprising. Subsequent studies indicated a multipronged antileukemic mechanism of PI3Kδ inhibition (reviewed in Vanhaesebroeck et al.)^6^ due to (1) a cancer cell–intrinsic impact, with CLL and follicular lymphoma being dependent on chronic B cell antigen receptor signaling in which PI3Kδ is a key mediator. This tonic B cell antigen receptor signaling, even without antigen stimulation, creates a constitutive survival signal that depends heavily on PI3Kδ activity. PI3Kδ inhibition also dampens signaling in the malignant B cells by a range of cytokines, chemokines, costimulatory molecules, and adhesion receptors; (2) blocking signals from the microenvironment that support malignant B cell survival and trafficking in their protective niche, by inhibition of stromal cells such as mesenchymal fibroblast-like cells, myeloid cells and leukemia-associated T cells, which together can lead to release of malignant B cells from their niches and increased vulnerability to combination agents, such as rituximab that was used in the CLL trial^14^; and (3) induction of a host antileukemia adaptive immune response, as a consequence of dampening of Treg function upon PI3Kδ inhibition.^17^ This break of immune tolerance also leads to autoimmune-like impacts, including colitis, which was also reported earlier in PI3Kδ kinase–dead mice.^7^

Over the next decade, several other PI3Kδ inhibitors were developed, an effort aided by the crystal structure of PI3Kδ reported in 2010.^18^ This study revealed conformational flexibility in the catalytic domain of p110δ, enabling inhibitors to engage with specific amino acid residues in p110δ outside the ATP-binding pocket, allowing design of inhibitors with PI3Kδ selectivity over other PI3K isoforms. Several inhibitors were approved for B cell malignancies, including duvelisib (dual PI3Kγ/δ inhibitor), umbralisib (PI3Kδ and casein kinase-1ε inhibitor), copanlisib (pan-class I PI3K inhibitor), and linperlisib (PI3Kδ inhibitor).

It is fair to say that the immunoregulatory impact of PI3Kδ inhibition was neither fully understood nor adequately appreciated during the early clinical studies with idelalisib. These immune effects were confounded by the administration of PI3Kδ inhibitors at maximum tolerated dose in a continuous administration scheme. This was further obscured by the enrollment in these early trials of heavily pretreated and/or elderly patients with reduced immune competency in which the adverse effects were mostly manageable. Indeed, in follow-up trials with idelalisib in treatment-naive and often younger patients, idelalisib was associated with more severe adverse effects that were treatment limiting. These included bacterial infections, opportunistic infections, and inflammatory and/or autoimmune toxicities, including colitis and pneumonitis, which may partially result from an overactive immune response in tissue locations exposed to microbial antigens. These serious side effects of idelalisib and other PI3K inhibitors in B cell malignancies led to black box warnings for idelalisib and duvelisib, withdrawal of accelerated FDA approvals, withdrawal or termination of phase 3 trials, and voluntary withdrawals from the market by companies for some or all indications.

Subsequent preclinical studies in mice showed that intermittent dosing of PI3Kδ inhibitors can achieve antitumor activity.^19^^,^^20^ Notably, continuous PI3Kδ suppression was not required in these experiments, as tumor growth inhibition was actually enhanced by an intermittent in vivo dosing schedule.^19^ These findings suggest that intermittent dosing could represent a promising approach for future therapeutic regimens.

Recent advances have brought more encouraging developments to PI3Kδ drug discovery and development.

The first is the approval of the PI3Kδ inhibitor leniolisib^21^ as monotherapy for activated PI3Kδ syndrome (APDS), a primary immune deficiency that predisposes to recurrent infections, bronchiectasis and leukemia.^22^ APDS is due to germline mutations in *PIK3CD*^23^^,^^24^ or *PIK3R1*.^25^ In fact, the first association of *PIK3CD* mutations in humans with immunodeficiencies was reported as early as 2006.^26^ In APDS, leniolisib is well tolerated and leads to normalization of immune defects and improvements in health-related quality of life, positioning it as a long-term therapeutic option for this syndrome.^22^ In 2018, several groups reported individuals with loss-of-function *PIK3CD* mutations (leading to inactive or absent p110δ), presenting with immunodeficiency associated with impaired T and B cell activation.^27–29^

A second advance is the discovery of a novel binding mode of PI3Kδ inhibitors, exemplified by roginolisib,^30^ which differs from previously reported PI3Kδ inhibitors.^31^ Indeed, despite binding at the orthosteric ATP site, roginolisib is noncompetitive with ATP and acts by stabilizing a catalytic C-terminal helix, locking the enzyme in an inactive conformation.^31^ It is tempting to speculate that this contributes to the high PI3Kδ isoform selectivity of this compound and the early data suggesting a promising clinical tolerability. Roginolisib may also lay the foundation for a new class of PI3Kδ inhibitors.

Last, PI3Kδ inhibition is now also being explored in immunotherapy for solid tumors, based on the finding that it can lead to immune activation in mice,^13^ now confirmed in human head and neck cancer^20^ and in CLL.^17^ Linperlisib has been tested in a phase 1 solid tumor basket trial, in which this compound has shown acceptable tolerability.^32^ Roginolisib monotherapy has also demonstrated promising antitumor activity in cutaneous and uveal melanoma patients who had progressed on PD-1/PD-L1 inhibitors.^33^ FDA fast-track designation for roginolisib was granted for uveal melanoma in 2023, with a registration trial for this indication now in progress (NCT06717126). A trial of roginolisib in metastatic non-small cell lung cancer, in combination with anti-PD1 with or without docetaxel, is also ongoing (NCT06879717). Interestingly, PI3Kδ inhibition is now also re-emerging in the B cell malignancy landscape, with a trial of roginolisib combined with venetoclax and rituximab recently initiated in CLL (NCT06644183).

Taken together, after several setbacks, a deeper understanding of PI3Kδ biology, the development of novel PI3Kδ inhibitors, and more mechanism-based clinical strategies may finally enable harnessing the therapeutic potential of PI3Kδ inhibition. The understanding that strong, sustained PI3Kδ inhibition may induce immune activation by altering immune cell balance suggests that PI3Kδ inhibitors—aside from specific cases such as APDS that may require continuous normalization of PI3Kδ signaling—may not be well suited for chronic full-dose use, underscoring the need for careful dose selection and intermittent dosing strategies.
